# Genes representing the stress-dependent component in arterial hypertension development

**DOI:** 10.18699/vjgb-25-139

**Published:** 2025-12

**Authors:** D.Yu. Oshchepkov, Yu.V. Makovka, I.V. Chadaeva, A.G. Bogomolov, L.A. Fedoseeva, A.A. Seryapina, M.P. Ponomarenko, A.L. Markel, О.Е. Redina

**Affiliations:** Institute of Cytology and Genetics of the Siberian Branch of the Russian Academy of Sciences, Novosibirsk, Russia; Institute of Cytology and Genetics of the Siberian Branch of the Russian Academy of Sciences, Novosibirsk, Russia; Institute of Cytology and Genetics of the Siberian Branch of the Russian Academy of Sciences, Novosibirsk, Russia Novosibirsk State University, Novosibirsk, Russia; Institute of Cytology and Genetics of the Siberian Branch of the Russian Academy of Sciences, Novosibirsk, Russia; Institute of Cytology and Genetics of the Siberian Branch of the Russian Academy of Sciences, Novosibirsk, Russia; Institute of Cytology and Genetics of the Siberian Branch of the Russian Academy of Sciences, Novosibirsk, Russia; Institute of Cytology and Genetics of the Siberian Branch of the Russian Academy of Sciences, Novosibirsk, Russia; Institute of Cytology and Genetics of the Siberian Branch of the Russian Academy of Sciences, Novosibirsk, Russia Novosibirsk State University, Novosibirsk, Russia; Institute of Cytology and Genetics of the Siberian Branch of the Russian Academy of Sciences, Novosibirsk, Russia

**Keywords:** rat, human, differentially expressed gene (DEG), arterial hypertension, stress, biomedical model, principal component analysis, крыса, человек, дифференциально экспрессирующийся ген (ДЭГ), артериальная гипертония, стресс, биомедицинская модель, метод главных компонент

## Abstract

Hypertension is among the major risk factors of many cardiovascular diseases. Chronic psychoemotional stress is one of its key causes. Studies of molecular mechanisms of human hypertension development are conducted in animals, including artificial rat strains that model various forms of the disease. The RatDEGdb database, used in our work, includes 144 hypothalamic genes that represent the common response to single short-term restraint stress in hypertensive ISIAH and normotensive WAG rats. These rat genes were annotated with changes in the expression of the human orthologs using data on 17,458 differentially expressed genes (DEGs) from patients with hypertension compared to normotensive subjects. We applied principal component analysis to orthologous pairs of DEGs identified in hypertensive patients and rat hypothalamic DEGs upon single short-term restraint stress. Two principal components, corresponding to a linear combination of log2 expression changes associated with the similarity (PC1) and difference (PC2) in the response to psychoemotional stress in two rat strains, on the one hand, and different forms of human hypertension, on the other, explained 64 % and 33 % of the variance in differential gene expression, respectively. The significant correlation revealed between PC1 and PC2 values for the group of DEGs with stress-induced downregulation indicates that psychoemotional stress and hypertension share a common molecular mechanism. Functional annotation suggests that stress-induced downregulation of genes involved in the plasma membrane function and, simultaneously, interactions with the extracellular matrix is the most likely contribution of psychoemotional stress to the development of the hypertensive status in patients, and the SMARCA4 transcription factor is the most likely mediator in the epigenetic modification affecting gene expression under chronic stress. Peripheral blood markers for the diagnosis of psychoemotional stress are proposed.

## Abstract (ru)

Гипертония считается ведущим фактором риска развития многих сердечно-сосудистых заболеваний. Одним из ключевых факторов, способствующих развитию гипертонии, является хронический психоэмоциональный стресс. Изучение молекулярно-генетических механизмов развития гипертонии человека проводят на животных, в том числе на специально созданных инбредных линиях крыс, моделирующих различные формы гипертонии человека. В настоящей работе использованы данные из базы данных RatDEGdb о 144 генах гипоталамуса, которые представляют общий ответ на рестрикционный стресс у гипертензивных крыс НИСАГ и нормотензивных крыс WAG. Эти гены крыс были аннотированы изменениями экспрессии ортологичных им генов человека с использованием данных о 17 458 дифференциально экспрессирующихся генах (ДЭГ) пациентов с артериальной гипертензией по сравнению с нормотензивными пациентами. Для выявленных пар ортологов между ДЭГ гипоталамуса крысы после рестрикционного стресса и пациентов с артериальной гипертензией применили анализ главных компонент. Две главные компоненты, соответствующие линейной комбинации значений log2 изменений экспрессии, связанные со сходством (PC1) и различием (PC2) ответа на психоэмоциональный стресс двух линий крыс, с одной стороны, и разными формами гипертонии человека, с другой, объясняли соответственно 64 и 33 % дисперсии дифференциальной экспрессии генов. Выявленная значимая корреляция между значениями PC1 и PC2 для группы ДЭГ со стресс-индуцированным снижением экспрессии указывает на существование общего молекулярного механизма между психоэмоциональным стрессом и гипертонией. Их функциональная аннотация позволила предположить, что стресс-индуцированное снижение экспрессии генов, участвующих в функционировании плазматической мембраны и одновременно во взаимодействии с межклеточным пространством, является наиболее вероятным вкладом психоэмоционального стресса в формирование гипертензивного статуса пациентов, а транскрипционный фактор SMARCA4 – наиболее вероятным участником эпигенетической модификации экспрессии генов в результате хронического стресса. Также предложены маркеры периферической крови для диагностики психоэмоционального стресса.

## Introduction

Arterial hypertension is a multifactorial disease. Its development is caused by both hereditary and environmental factors. It is contributed by chronic psychoemotional stress, which can be induced by occupational factors, social isolation, low socioeconomic status, anxiety, distress, and other lifestyle factors (Liu M.Y. et al., 2017).

Chronic stress factors hyperactivate the sympathetic system. This process is accompanied by neuroinflammation and mitochondrial dysfunction. In the brain, it may result in the accumulation of reactive oxygen species (ROS), which exceed the neuronal antioxidant capacity (Lambert A.J., Brand, 2009; Hovatta et al., 2010; Picard, McEwen, 2018). Oxidative stress triggers the cell membrane damage cascade, enhances lipid peroxidation, and impairs neural conduction (Che et al., 2015; Montezano et al., 2015). Neuron damage affects the activity of neuronal circuits; in particular, it reduces the GABAergic inhibitory activity and results in the predominance of glutamatergic exciting signals in sympathetic nuclei. This enhances impulse transmission in the cardiovascular system and causes persisting blood pressure (BP) increase (Lambert E.A., Lambert G.W., 2011; Hering et al., 2015). The emerging closed cycle of neurogenic and oxidative stresses promotes the formation of a pathological vegetative pattern, in which even minor stressful events cause pronounced BP increase (Fontes et al., 2023)

Thus, not only hereditary predisposition can cause hypertension. Some patients develop hypertension due to a combination of other causes. Their multitude hampers the understanding of underlying molecular processes and determination of causes that are associated with hereditary predisposition. Hereditary causes may include high stress sensitivity; other processes may be triggered by, e. g., elevated salt sensitivity or related to endothelial or endocrine dysfunction. Knowledge of these processes may be helpful in seeking approaches to the pathogenetic therapy of hypertensive disease. Our study is focused on this issue, in particular, the identification of the stress-associated component of hypertension development.

Strains of hypertensive rats characterized by a broad range of pathophysiological changes in the cardiovascular system are often used in studies of the molecular mechanisms of hypertension. Each of such strains models a certain form of arterial hypertension. One of them is ISIAH (Inherited Stress- Induced Arterial Hypertension), prone to stress-induced arterial hypertension. It models spontaneously developing hypertension, marked by severe response (BP increase) to psychoemotional stress (Markel, 1992; Markel et al., 1999, 2007).

Stress adaptation in cells involves a significant remodeling of gene expression programs (de Nadal et al., 2011). However, the molecular mechanisms underlying stress adaptation are still poorly understood. Formerly, we showed that the transcription levels of many genes in the hypothalamus of hypertensive ISIAH and normotensive WAG/GSto-Icgn (Wistar Albino Glaxo, hereinafter WAG) rats changed when the animals were exposed to short-term (2 h) restraint stress in tight wire-mesh cages. These changes may affect a great number of biologic processes and metabolic pathways (Oshchepkov et al., 2024). The focus on hypothalamic genes is due to the fact that this brain region is among the key ones regulating the neuroendocrine response to stress. It integrates the central and peripheral components involved in blood pressure regulation and arterial hypertension development by controlling glucocorticoid secretion (Carmichael, Wainford, 2015; Burford et al., 2017; Kinsman et al., 2017; Fontes et al., 2023). Actually, it is a key link between stress and hypertension development. The studied stress model induced a significant BP increase in ISIAH but not in WAG rats, although blood corticosterone levels significantly increased in both rat strains; thus, both hypertensive and normotensive animals responded to stress (Oshchepkov et al., 2024). Therefore, we presume that the differentially expressed genes (DEGs) associated with the common (hypertension genotype-independent) response of the rat hypothalamus on restraint stress (considered psychoemotional)are involved in the stress-dependent componen.

In human daily routine, stress can be induced by many factors to activate the sympathetic nervous system and cause hypertension, as well as other cardiovascular diseases. As we mentioned, the hypothalamus takes a significant part in these processes. The study of molecular mechanisms of hypothalamic response to stress in humans is difficult, and there is no relevant information on gene expression in brain regions in hypertensive patients. Nevertheless, PubMed presents commonly available independent sets of experimental data on gene expression in other organs and tissues, including peripheral blood, in hypertensive patients and in biomedical cellular models of hypertension (Oshchepkov et al., 2022; Shikhevich et al., 2023). Here we employ these data to reveal human genes orthologous to rat genes associated with the response to restraint (psychoemotional) stress and to identify genes forming the stress-dependent component in hypertension development in the human. 

## Materials and methods

Experimental animals. Experiments were conducted with 3-month old male rats of the hypertensive ISIAH and normotensive WAG strains in the conventional vivarium of the Center for Experimental Animal Genetic Resources, Institute of Cytology and Genetics, Novosibirsk, Russia. The animals were kept under standard conditions at the light schedule 12:12. Water and standard diet were given ad libitum.

Transcriptome analysis in the hypothalamus by the RNASeq method was done in four groups of seven animals each: (1) ISIAH_control, (2) WAG_control, (3) ISIAH_stress, and (4) WAG_stress. Basal systolic arterial BP was measured by the tail-cuff method (Markel et al., 2007). Rats were seminarcotized with ether to avoid emotional stress during the measurement. The experimental rats in ISIAH_stress and WAG_stress groups were exposed to restraint (emotional) stress seven days after BP measurement. In this procedure, an animal was placed into a tight wire-mesh cage for 2 h; for details, see (Oshchepkov et al., 2024).

All international guidelines for the care and use of laboratory animals were followed. Animal protocols were approved by the ICG Bioethics Committee, Novosibirsk, Russia, protocol No. 115 of December 20, 2021.RNA-Seq. RNA was isolated at the Institute of Genomic Analysis, Moscow, Russia. Hypothalamus sample preparation and transcriptome sequencing were conducted at BGI Hongkong Tech Solution NGS Lab. following manufacturer’s recommendations (MGI Tech Co., Ltd., China). Paired-end sequencing of cDNA libraries was performed by DNBSEQ Technology with read length 150 bp and sequencing depth over 30,000,000 uniquely mapped reads. All samples were analyzed as biological replicates.The sequencing results were preprocessed with FastQC software version 0.11.5 (Andrews, 2010) to check the quality. The overall number of nucleotide reads in the libraries after the preprocessing was 1,287,393,367; of them, 1,267,436,623 (98.45 %) were mapped on the reference rat genome mRatBN7.2/ rn7 (rn7 assembly, Wellcome Sanger Institute Nov, 2020) with STAR 2.7.10a software (Dobin et al., 2013).

The mapping data were statistically processed to calculate differential expression of genes in the R environment for statistical computing. We applied surrogate variable analysis SVA (Leek et al., 2012) to take into account undesirable variations in data caused by inadvertent systematic deviations during sample preparation. Prior to SVA, the expression data were normalized and transformed with the vst function in DESeq2 v1.30.1 (Love et al., 2014) according to online documentation. Relevant surrogate variables were then included as factors in the differential expression analysis with DESeq2. The differential expression analysis was conducted separately for each pair of groups.

Differential expression was calculated for all genes exhibiting significant expression levels above the threshold: sum of gene coverages in all libraries over 10 reads. The significance level for DEG detection was chosen with the consideration of the correction for multiple comparisons. It corresponded to adjusted p-value < 5 % and Log2 fold change ≥ |0.585| (1.5 fold). The information on the revealed DEGs had been described in (Makovka et al., 2024; Oshchepkov et al., 2024) and uploaded to the RatDEGdb database (Chadaeva et al., 2023). The data are presented as transcription upon stress normalized to transcription at rest

Choice of human genes whose expression changes in hypertension. We used generally accessible sets of experimental data on patients with hypertensive disease and data on cellular hypertension models available from PubMed (Lu, 2011). The sample included only data reported as statistically significant according to Fisher’s Z test with correction for multiple comparisons (PADJ <0.05). We selected 16 publications with data on 17,458 genes differentially expressed in tissues and cells of hypertensive and normotensive subjects. This list of DEGs presents 16 tissues and 15 hypertension forms (Table 1). The threshold for a significant change in transcription level was set to be 1.5 times. The data are presented as the transcription level in hypertensive patients normalized to normotensive subjects.

**Table 1.**
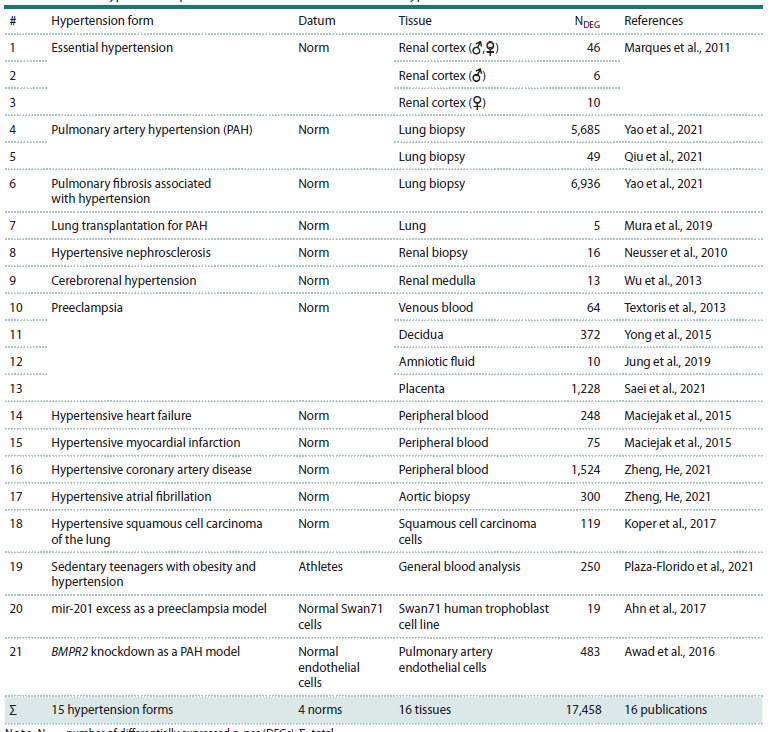
DEGs in hypertensive patients and biomedical cell models of hypertension found in PubMed Note. NDEG, number of differentially expressed genes (DEGs); Σ , total

Bioinformatical analysis of orthologous genes in the rat and human. Orthologs were sought with the Gene and Ortholog Location Finder (GOLF) module in the Rat Genome database (https://rgd.mcw.edu/rgdweb/ortholog/ start.html) (Vedi et al., 2023). Pairwise combinations of log2 changes in the expression of orthologous genes were analyzed by the principal component method with the conventional Past v.4.04 software (Hammer et al., 2001). Two values corresponded to log2 expression changes for ISIAH and WAG rats and one for the human orthologous gene. The same software was applied to the calculation of factor loadings and the statistical significance of the explained portion of component variance from 1,000 bootstrap samples (Efron et al., 1996). Pearson correlation analysis was conducted with the two-tailed test of significance. Groups of gene pairs for which the first principal component PC1 was above zero and groups for which it was below zero were analyzed separately.

Functional annotation of DEGs. Functional enrichment networks were constructed with the STRING database (Szklarczyk et al., 2023). The functional annotation of DEGs was done with Enrichr-KG (Evangelista et al., 2023). Analysis of the enrichment of DEG promoter regions with transcription factor binding sites was done with Enrichr (Xie et al., 2021).

## Results

We studied genes that change their transcription levels by more than 1.5 fold in the hypothalamus of hypertensive ISIAH and normotensive WAG rats upon short-term (2 h) restraint stress. There were 257 and 229 such genes, respectively. Of them, 144 DEGs produced common responses, 113 DEGs showed significant expression changes only in the ISIAH hypothalamus, and 85 genes significantly change their expression only in the WAG hypothalamus (Oshchepkov et al., 2024). To reveal genes that might form the stress-sensitive component in human hypertension development, we sought orthologous human genes differently expressed in subjects with hypertension and normal arterial blood pressure.

**Analysis of the common response**

The search for orthologs of the 144 rat DEGs in the GOLF Rat Genome Database and their comparison with the list of human DEGs found in PubMed (see Materials and methods) revealed 96 orthologous pairs. This set included data on six tissues of nine hypertension forms from seven publications (Supplementary Table S1, 96 common DEGs)1.

Supplementary Materials are available in the online version of the paper: https://vavilovj-icg.ru/download/pict-2025-29/appx54.xlsx


**Fig. 1.**
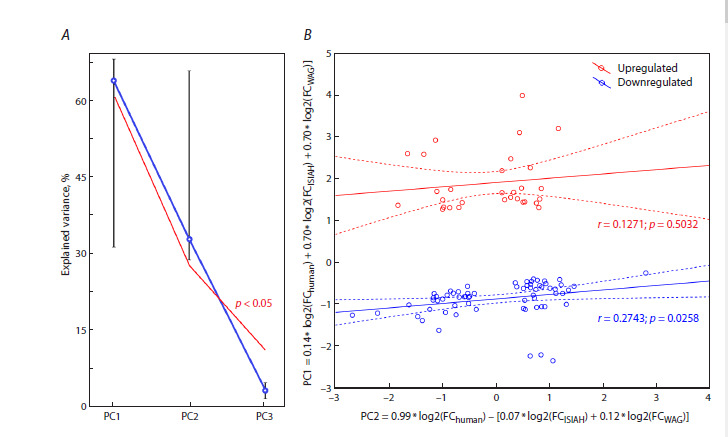
Factor analysis of differentially expressed genes (DEGs) in hypertensive subjects (Table 1) and stressed rats. A, choice of statistically significant (p < 0.05) principal components PC1 and PC2 in the factor analysis of DEGs in hypertensive subjects and stressed rats. Designations: ○, mean; whiskers, standard error of the mean; red line, lower boundary of the 95 % confidence range of the statistical significance of the explained variance. B, principal component and regression analysis. Designations: PC1 and PC2, the first (Y-axis) and second (X-axis) principal components as the corresponding linear combinations of original variables with regard to the calculated factor loadings. Red marks (Upregulated): group of DEGs with stress-induced upregulation; blue marks (Downregulated): group of DEGs with stress-induced downregulation according to the PC1 estimate. Dashed lines: boundaries of the 95 % confidence range for the regression line (solid). Letters r and p: linear correlation coefficients and their statistical significance, respectively, assessed with conventional Statistica (StatsoftTM, USA).

In succession to our earlier papers on the factor analysis of DEGs (Chadaeva et al., 2021; Shikhevich et al., 2023), we processed data on the changes in the expression of rat genes upon stress regardless of the hypertensive genotype and data on orthologous genes of hypertensive humans by the principal component method. As seen in Figure 1A, the 96 pairs of human genes and their rat orthologs are characterized by two principal components: PC1 and PC2. They explain 64 and 33 % of the variance, and both are statistically significant according to 1,000 bootstrap samples (Efron et al., 1996).

The results of the principal component analysis are shown in Figure 1B. We see that PC1, plotted along the Y-axis, is proportional to a linear combination of the original variables with regard to the calculated factor loadings. The combination summarizes the log2 values of changes measured in independent experiments with hypertensive subjects and log2 values of changes in rats of the two strains exposed to a single restraint stress. Thus, this component may characterize the similarity between the responses of two rat strains on psychoemotional stress, on the one side, and different forms of human hypertension, on the other side

The second principal component PC2, plotted along the X-axis, is proportional to a linear combination of the original variables with regard to the calculated factor loadings. The combination corresponds to the positive contribution of values obtained in independent experiments on humans and negative contribution of stress response values in the two rat strains. It may characterize differences between the species and features of particular impairments in hypertensive human patients. Figure 1B clearly shows that all DEGs studied are divided into two disjoint groups with respect to PC1 = 0. Red and blue colors mark genes that respond to stress by expression increase (Upregulated) and decrease (Downregulated), respectively. Regression analysis demonstrates a significant (p< 0.05) correlation between PC1 and PC2 in a group of 66 pairs of 52 DEGs in the Downregulated cluster (Fig. 1B, blue marks). This correlation may be indicative of a common molecular mechanism of gene suppression in the formation of stress response in the rat and a similar process in the human, which leads to hypertension.

The corresponding analysis of the group of DEGs with stress-induced upregulation (Upregulated) shows no significant correlation. Thus, the mechanisms activating this group of genes may be different in different tissues and disorder forms.

**Functional annotation of DEGs associated with stress-induced expression downregulation**

To understand the molecular mechanism downregulating genes in stress response formation common to the human and rat, we performed functional annotation of 52 DEGs associated with stress-induced downregulation (the Downregulated cluster in Fig. 1B). Eight genes associated with hypertension were found among the 52 genes of the Downregulated cluster: ALOX12, ATP2B4, CX3CR1, GRK3, KDR, NOS1, RASGRP3, and SMAD9. Five of them (ALOX12, GRK3, KDR, NOS1, and RASGRP3) showed unidirectional changes in the rat and human in all cases (Table S1, 96 common DEGs).

**Fig. 2.**
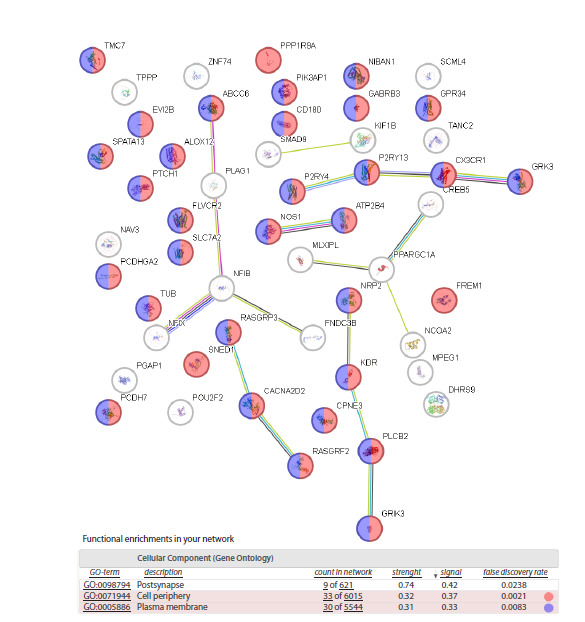
Screenshot of the annotation of the Downregulated cluster in Fig. 1B with the STRING database (Szklarczyk et al., 2023). Designations follow the STRING annotation.

Analysis of DEG enrichment in the STRING database. Analysis of the group of 52 DEGs in STRING revealed the most enriched terms: Postsynapse, Cell periphery, and Plasma membrane (Fig. 2). Thus, the process bringing these DEGs together is associated with extracellular matrix and the membrane, directly involved in its interaction with the cell.

Analysis with the Enrichr-KG resource. We analyzed biologic processes and metabolic pathways with the Enrichr- KG resource (Table 2). Signal Transduction was the most enriched term. It included 17 DEGs. This group included several hypertension-associated genes. The most enriched metabolic pathways were Adrenergic signaling in cardiomyocytes, Salivary secretion, Aldosterone synthesis and secretion, Glucagon signaling pathway, Insulin resistance, Differentiation of white and brown adipocyte, Constitutive Androstane Receptor Pathway, and Neovascularisation processes. They involve several DEGs under consideration associated with hypertension (Table 2). Some DEGs associated with the biological processes and metabolic pathways listed in Table 2 were found in peripheral blood.

**Table 2.**
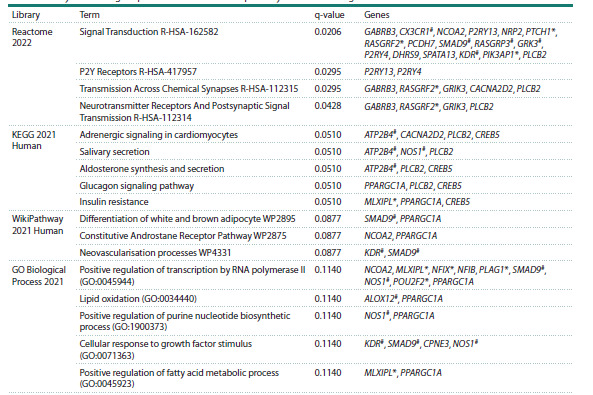
Analysis of biological processes and metabolic pathways of the Downregulated cluster * DEGs found in peripheral blood, # genes associated with hypertension.

**DEGs in peripheral blood in the Downregulated cluster**

We found 14 DEGs belonging to the Downregulated cluster in the peripheral blood of patients (Table 3). These DEGs are promising as candidate peripheral markers associated with psychoemotional stress

**Table 3.**
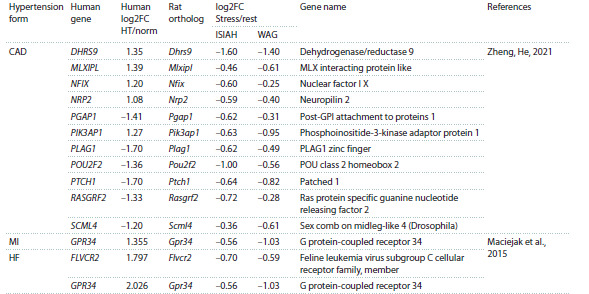
Peripheral blood markers in the Downregulated cluster Note. CAD, hypertension-induced coronary artery disease; MI, hypertension-induced myocardial infarction; HF, hypertension-induced heart failure; HT/norm, transcription level in hypertensive subjects normalized to the transcription level in subjects with normal arterial blood pressure

**Analysis of transcription factors involved in the regulation of DEGs of the Downregulated cluster**

Analysis of DEGs of the Downregulated cluster with the Enrichr resource revealed transcription factors that may be involved in the regulation of these DEGs in the rat hypothalamus and in tissues of hypertensive patients (Table 4). The most enriched terms in the ChEA_2022 library are associated with transcription factors SMARCA4 (SWI/SNF related BAF chromatin remodeling complex subunit ATPase 4) and glucocorticoid receptor NR3C1 (nuclear receptor subfamily 3 group C member 1). Receptor NR3C1 and six transcription factors (PPARG, ESR1, AR, NFE2L2, BRD4, and STAT3) presented in Table 4 are associated with hypertension.

**Table 4.**
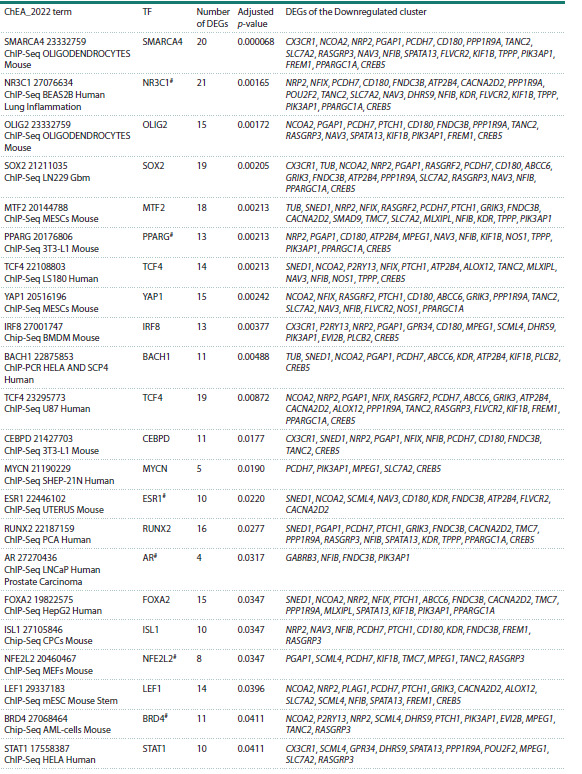
Analysis of transcription factors involved in the regulation of DEGs of the Downregulated cluster # Transcription factors encoded by genes associated with hypertension; TF, transcription factor

**Table 4end.**
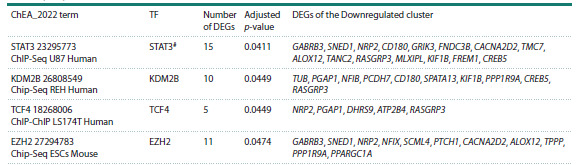
Table4end.

## Discussion

The hypothesis of an association between chronic psychosocial stress and hypertension was put forward long ago (Spruill, 2010). It has been confirmed in several studies (Liu M.Y. et al., 2017; Bautista et al., 2019), but the molecular mechanisms responsible for this association remain obscure.

Here we considered 144 genes for common stress response in the hypothalamus of hypertensive and normotensive rats and compared this response with changes in gene transcription levels in different organs and tissues in patients with different hypertension forms. It follows from our results that the transcription changes observed may point to some common features in the restructuring of the gene expression pattern, which reflect the contribution of psychoemotional stress to the manifestation of the hypertensive condition.We found human DEGs orthologous to 144 genes that change their transcription in response to single short-term restraint stress in the hypothalamus of hypertensive ISIAH and normotensive WAG rats. Analysis by the principal component method revealed two DEG clusters and demonstrated a significant correlation between PC1 and PC2 values for the DEG cluster with stress-induced downregulation. This correlation suggests the existence of a molecular mechanism suppressing gene expression in the formation of stress response shared by the human and rat. It is in good agreement with the notion that adaptation to stress is accompanied by remodeling of the functional transcriptome and arrest of translational processes (Advani, Ivanov, 2019; Baymiller, Moon, 2023). It should be mentioned that transcription up- or downregulation under stress can be both compensatory and pathogenetic, as can be elucidated by functional analysis of a particular gene. Gene downregulation can stimulate many processes, contributing to the activation of the sympathetic nervous system and enhancing oxidative stress in tissues. It may also participate in neuroprotection, as we discussed earlier in connection with some DEGs (Makovka et al., 2024; Oshchepkov et al., 2024).

The enrichment in the linked terms Plasma membrane and Cell periphery found in the group with stress-induced downregulation encompasses 30 and 33 genes of 52, respectively. It is consistent with the idea that the change in the composition of extracellular matrix associated with the release of corticosterone and catecholamines resulting from the activation of the hypothalamic–pituitary–adrenal (HPA) axis and the sympathetic nervous system is the most common feature in the model of human chronic stress. This change triggers various signaling pathways, including free radical generation, apoptosis, elevated glutamate release in synapses, and production/release of cytotoxic cytokines (Bali et al., 2013). These intercellular signals, in turn, may affect gene expression, leading to structural and functional changes in plasma membranes (Bali et al., 2013), such as changes in the lipid profile of platelet membranes (Bikulciene et al., 2024) and to neuron excitability due to modifications in membrane structure and composition (Rosenkranz et al., 2010; Matovic et al., 2020), including the postsynaptic membrane, as indicated by the enrichment of the term Postsynapse in our study. Analysis of biological processes and metabolic pathways in the DEG cluster with stressinduced downregulation also emphasizes the role of signal transduction and postsynaptic signal transmission (Table 2). Stress can also impair the expression of genes for autophagy marker proteins and disturb the concentrations of lysosomal proteins and enzymes (Ulecia-Moron et al., 2025). Generally speaking, the considerable number of genes downregulated in response to stress that are common in different organs and tissues characterizes the scale of changes affecting the plasma membrane in response to stress-induced release of active signaling molecules into the extracellular matrix.

The DEG cluster with stress-induced downregulation was found to include eight hypertension-associated genes. Their expression changes may influence biological processes and metabolic pathways involved in blood pressure regulation: Adrenergic signaling in cardiomyocytes (Maltsev et al., 2019), Aldosterone synthesis and secretion (Ferreira et al., 2021), and Lipid oxidation (Leong, 2021).

Our study revealed genes encoding transcription factors that may be involved in the regulation of genes of the DEG cluster with stress-induced downregulation. We suppose that SMARCA4 (SWI/SNF-related BAF chromatin remodeling complex subunit ATPase 4) and NR3C1 (nuclear receptor subfamily 3 group C member 1, the glucocorticoid receptor) are essential for the processes.

SMARCA4 belongs to the family of proteins with helicase and ATPase activities. These proteins play the key role in the arrangement of chromatin conformation, which is crucial for gene regulation. The SWI/SNF (BRG1) complex has been shown to participate in the epigenetic and transcription regulation in vascular smooth muscle cells, thus being associated with the development of cardiovascular disorders (Liu H. et al., 2024). Genetic polymorphisms in the SMARCA4 gene are associated with hypertension risk (Ma et al., 2019). The SWI/ SNF complex can be directly recruited by glucocorticoid receptor NR3C1 for remodeling chromatin in glucocorticoiddependent gene activation to potentiate the glucocorticoid receptor action afterwards (Fryer, Archer, 1998; Wallberg et al., 2000). This consideration is in line with the inference by Patel et al. (2025) that SMARCA4 may be the key regulator in glucocorticoid-induced increase in intraocular tension (regarded as ocular hypertension by the authors), which can result in secondary glaucoma

Glucocorticoid receptor NR3C1, upregulated by steroid hormones, is associated with hypertension. It acts as a transcription factor (Timmermans et al., 2019). Our previous study demonstrated Nr3c1 downregulation in the hypothalamus of both rat strains under stress: log2FC = −0.133 in ISIAH and log2FC = −0.113 in WAG. The decrease in glucocorticoid receptor activity after the increase in glucocorticoid level upon stress can be interpreted as regulation of the homeostasis of glucocorticoid receptors (Burnstein et al., 1991). The results of this work suggest the involvement of NR3C1 in the regulation of 21 genes associated with the DEG cluster with stress-induced downregulation. The fact that the lists of target genes for NR3C1 and SMARCA4 intersect significantly (14 common targets in Table 4, p- value = 0.0007) agrees well with the aforementioned data that the regulation networks of these TFs are interrelated and, correspondingly, many genes we found can undergo SMARCA4-mediated epigenetic modification under stress, whose key signal is NR3C1 activation.

Our approach to data analysis revealed 14 DEGs in peripheral blood (Table 3). The genes listed in Table 3 have not been found to be associated with hypertension thus far, but our study suggests that they may be candidate peripheral markers associated with psychoemotional stress in hypertensive patients. Our results are insufficient for a reliable choice of peripheral blood markers suitable for clinical practice. This issue demands a special study aimed at the corroboration of the role of the markers in the blood of patients with chronic hypertension. Such a study would also help in determining their reference values in clinical use.

## Conclusion

Our work revealed stress-induced downregulation of genes involved in plasma membrane function and, simultaneously, in interaction with the extracellular matrix. This downregulation reflects the significant contribution of psychoemotional stress to the formation of hypertension in humans and appears to be a fundamental process linking chronic stress and hypertension, primarily in the hypothalamus, which is a key component of this link and the focus of our study. Numerous publications confirm the effect of stress on the plasma membrane. This process is universal, even when comparing stress responses in the rat and fruit fly (Podkolodnaya et al., 2025). Furthermore, according to the membrane concept of arterial hypertension pathogenesis (Orlov, 2019), changes in membrane structure and permeability are among the chief processes underlying the impairments that promote the development of hypertensive disease. The identified target genes of the SMARCA4 transcription factor, which is likely to be involved in their epigenetic regulation, are the most probable common factor mediating long-term alteration of gene expression patterns caused by chronic stress. Its further investigation looks promising. On the grounds of our data, we propose candidate peripheral blood markers that may be helpful in clinical practice to diagnose psychoemotional stress.

## Conflict of interest

The authors declare no conflict of interest.
